# Room-temperature carbon monoxide oxidation by oxygen over Pt/Al_2_O_3_ mediated by reactive platinum carbonates

**DOI:** 10.1038/ncomms9675

**Published:** 2015-10-22

**Authors:** Mark A. Newton, Davide Ferri, Grigory Smolentsev, Valentina Marchionni, Maarten Nachtegaal

**Affiliations:** 1 Department of Physics, University of Warwick, Gibbet Hill Road, Coventry CV4 7AL, UK; 2 Paul Scherrer Institut, CH-5232 Villigen, Switzerland

## Abstract

Room-temperature carbon monoxide oxidation, important for maintaining clean air among other applications, is challenging even after a century of research into carbon monoxide oxidation. Here we report using time-resolved diffuse reflectance infrared spectroscopy, X-ray absorption fine structure spectroscopy and mass spectrometry a platinum carbonate-mediated mechanism for the room-temperature oxidation of carbon monoxide. By applying a periodic reduction–oxidation mode of operation we further show that this behaviour is reversible and can be formed into a catalytic cycle that requires molecular communication between metallic platinum nanoparticles and highly dispersed oxidic platinum centres. A new possibility for the attainment of low-temperature oxidation of carbon monoxide is therefore demonstrated.

## Abstract (web summary)

There is economic impetus to achieve low-temperature carbon monoxide oxidation. Here, the authors use time-resolved diffuse reflectance infrared spectroscopy and mass spectrometry to show that platinum carbonates formed reversibly from platinum/aluminium oxide can oxidize carbon monoxide at room temperature.

## Graphical abstract

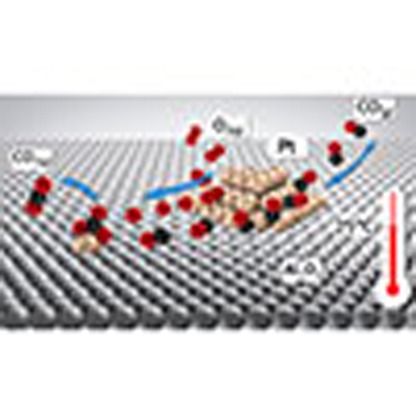

The catalytic oxidation of CO by O_2_ by platinum is one of the longest established and industrially important heterogeneous catalytic conversions. The generally accepted Langmuir–Hinshelwood (LH) mechanism requires the adsorption and reaction of molecular CO with atomic oxygen over metallic platinum surfaces^1^,^2^,^3^,^4^,^5^. At temperatures where adsorbed molecular CO becomes stable, the catalytic cycle cannot be completed as the Pt surfaces become poisoned by adsorbed CO, which prevents dissociation of O_2_. Catalysis therefore becomes efficient only when the steady-state coverage of CO is diminished to the degree where dissociation of O_2_ can efficiently occur. Practically speaking, this restricts effective CO oxidation using Pt-Al_2_O_3_ catalysts to temperatures >400 K (Supplementary Fig. 1 shows this for a conventional test of catalytic light off using the 5 wt% Pt/Al_2_O_3_ used in this study). However, Pt remains the metal of choice in many applications for effective CO conversion to CO_2_ where sufficient temperatures are intrinsic or can be easily applied.

There are strong drivers, be they economic or regulatory in nature, to achieve CO oxidation at lower, ideally ambient, temperatures. This is especially the case in the purification of fuel (H_2_) feeds, emission control and the maintenance of clean air. Given the fundamental limitations of the LH mechanism for simple Pt catalysts, research to achieve low-temperature CO oxidation has proceeded in other directions: Pt may be favourably modified through contact with more exotic oxides, for instance CeO_2_ (refs 6, 7) and, most recently, iron–nickel hydroxides^8^, or other metals, most notably Au, may be employed to achieve low-temperature CO conversion^9^,^10^,^11^.

Beyond the classic LH mechanism, some attention has recently been drawn to reactive Pt surface oxides and carbonates in CO conversion^12^,^13^,^14^,^15^,^16^,^17^. Ackermann *et al.*^12^ determined the presence of two reactive, single-layer surface oxides formed on Pt(110). The incommensurate oxide (essentially a sheet of hexagonal PtO_2_) has been latterly observed during CO oxidation over Al_2_O_3_-supported Pt nanoparticles^13^. The second reactive and commensurate (1 × 2) surface oxide observed by Ackermann *et al.*^12^ has yet to be directly observed on high surface area nanosized catalysts. Intriguingly, however, calculations^12^ suggested that this oxide requires the stabilization of an additional carbonate species.

Most recently, Moses-DeBusk *et al.*^17^ have theoretically constructed an entire catalytic cycle that converts CO to CO_2_ solely through isolated Pt centres, adsorbed on Al_2_O_3_ (010) surfaces, which requires the involvement of reactive carbonates. The existence of such Pt carbonates on low loaded (0.18 and 1 wt%) Pt/Al_2_O_3_ catalysts was verified using diffuse reflectance infrared Fourier transform (DRIFTS), although their intrinsic reactivity was not addressed.

Herein, we reveal a route to CO_2_ that is mediated through such Pt carbonates and active at ambient temperature. This is achieved using a commercially available catalyst system comprising 5 wt% Pt (average particle diameter 3 nm, see Supplementary Fig. 5) supported on a majority *θ*-Al_2_O_3_ co-existing with some *γ*-Al_2_O_3_ phase (see Supplementary Fig. 4). We show, using periodic redox operation, that this route to CO_2_ can be made catalytic, and that achieving such reversibility requires a synergy between metallic Pt nanoparticles and non-metallic Pt centres that are the precursors to the carbonates. A new mechanism for low-temperature CO conversion using Pt/Al_2_O_3_-based catalysts is therefore demonstrated.

## Results

### Periodic CO oxidation of the 5 wt% Pt/Al_2_O_3_ catalyst at 298 K

**Figure 1:**
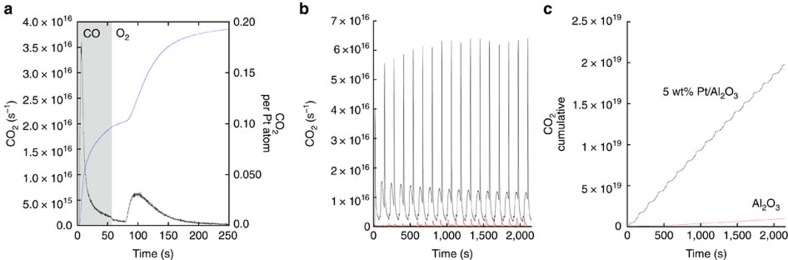
Periodic redox operation of the 5 wt% Pt/Al_2_O_3_ catalyst at 298 K. (**a**) Transient evolution of CO_2_ observed during exposure of a pre-reduced Type-94 (Johnson Matthey) 5 wt% Pt/Al_2_O_3_ catalyst to 5 vol% CO/Ar (shaded area), followed by a switch to 21 vol% O_2_/He at 298 K. The left-hand axis reports the evolution of CO_2_ in terms of molecules per second (black), the right-hand axis shows the cumulative CO_2_ production as a fraction of the total number of Pt atoms in the catalyst bed (blue). (**b**) Repeated cycles of a similar (shorter oxidizing cycle) experiment shown in **a**: black=5 wt% Pt/Al_2_O_3_; red=Al_2_O_3_. (**c**) Cumulative CO_2_ (molecules) production during the experiment shown in **b**.

Figure 1a shows variations in the CO_2_ production measured by mass spectrometry (MS) during room-temperature exposure of a 5-wt% Pt/Al_2_O_3_ (Type-94, Johnson Matthey) to 5 vol% CO/Ar (50 ml min^−1^). Supplementary Fig. 3 shows equivalent data for a second commercial catalyst (2 wt% Pt/γ-Al_2_O_3_, Umicore), whereas Supplementary Fig. 4 compares both catalysts from the perspective of X-ray diffraction, see also Supplementary Discussion. The feed is then switched to 21 vol% O_2_/Ar at the same flow rate. This sample had been previously reduced *in situ* (5 vol% H_2_/Ar to 573 K) before being returned to an Ar flow before the subsequent CO/O_2_ exposure cycle at 298 K. Figure 1b shows the CO_2_ production resulting from extending the experimental protocol of Fig. 1a to a periodic operation over repeated CO/O_2_ switches. Alongside the CO_2_ production from the 5 wt% Pt/Al_2_O_3_ the red curve shows that obtained from an unloaded Al_2_O_3_ sample (Condea Puralox (γ)). Finally, Fig. 1c shows the cumulative CO_2_ production achieved over the duration of the periodic operation of Fig. 1b.

CO_2_ is produced instantaneously on the admission of CO before rapidly returning towards baseline levels within the cycle. At the same time (not shown), an exotherm (*ca*. 3 K) is also transiently observed. On O_2_/Ar admission, CO_2_ is again observed to be formed but with some delay from the switching out of CO in favour of O_2_. The integrals show that in each branch of the redox switch practically the same number of CO_2_ molecules are produced, indicating a quantitative reversibility. This reversibility is confirmed (Fig. 1b,c) by repeated gas switching and is compared with a similar experiment carried out over a Pt-free Al_2_O_3_ (red lines). The total number of CO_2_ molecules produced over the first cycle shown in Fig. 1a is equivalent to almost 0.2 CO_2_ per Pt atom present in the bed. This implies that in each half of the first cycle the active phase comprises only *ca*. 10% of the Pt. Figure 1b shows that the CO_2_ production in the CO cycle actually improves significantly after the first cycle (Fig. 1a) and is maintained at a higher level thereafter; as such, the 10% estimate of the active fraction of the Pt represents a lower limit, as subsequent cycles might indicate that a range of 10–20% may be appropriate. Over the extended period of the experiment (Fig. 1c), the total amount of CO_2_ produced is some five times the number of Pt atoms present in the catalyst bed.

### DRIFTS during periodic CO oxidation operation at 298 K

**Figure 2:**
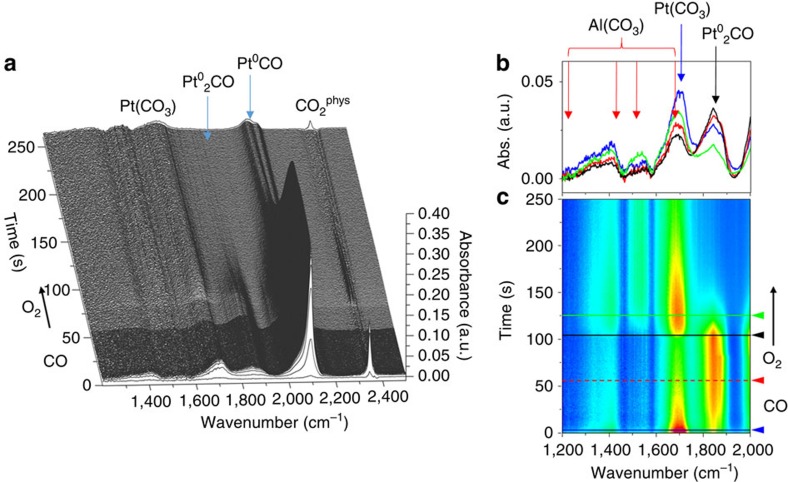
DRIFTS during periodic redox operation at 298 K. (**a**) DRIFTS spectra derived from a first CO/O_2_ cycle shown in Fig. 1. (**b**) The 1,200–2,000 cm^−1^ region of the DRIFTS shown to emphasize the reactive behaviour of species below *ca*. 1,750 cm^−1^ as compared with the bridging 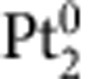
CO (*ca*. 1,850 cm^−1^). (**b**) Individual absorbance spectra corresponding to the different arrow/lines shown in **c**. The band positions expected for different aluminum carbonates (red), Pt(CO_3_) (blue) and 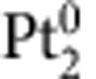
CO (black) are also given^21^,^22^. (**c**) Colour map representation of the same set of DRIFTS spectra of **a**. The red arrow shows the changeover point in time between the CO/Ar flow and the O_2_/Ar flow; the solid black line highlights the delay between the switch to O_2_, the removal of bridging 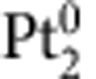
CO species and the transient re-appearance of the bridging 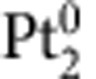
CO band at *ca*. 1,700 cm^−1^.

Figure 2a shows the evolution of infrared bands during the single CO/O_2_ cycle of Fig. 1a. Figure 2c shows a colour map of the 1,200–2,000 cm^−1^ region of the DRIFTS spectra to emphasize the temporal behaviour of infrared-active species in this region.

**Figure 3:**
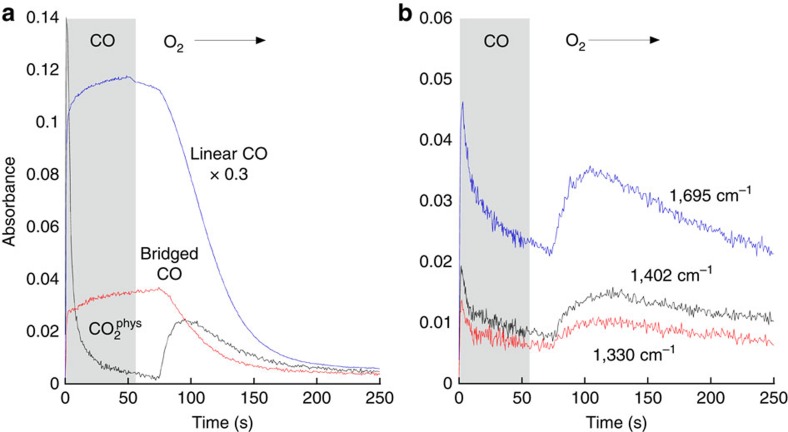
Temporal evolution of adsorbed species at 298 K. (**a**) Linear CO (blue: 2,094 cm^−1^), bridged CO (red: 1,845 cm^−1^) and 
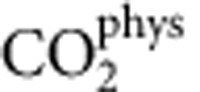
 (black: 2,345 cm^−1^). (**b**) Other bands at 1,695, 1,402 and 1,330 cm^−1^ as indicated. The shaded area indicates the period of exposure to 5% CO.

Figure 3a,b shows the temporal variations in a number of different infrared-visible bands observed in Fig. 2 and how they relate to the observed production of CO_2_. As might be expected, under CO the DRIFTS spectra are dominated by bands due to linear (2,094 cm^−1^) and bridged CO (1,845 cm^−1^) adsorbed on metallic Pt^18^. These appear instantaneously on introduction of CO and persist beyond the introduction of O_2_ before attenuating to any significant degree. Alongside these bands a number of other transient species are also seen, to rapidly evolve and then attenuate. The largest of these under CO is a sharp feature at 2,345 cm^−1^ due to physisorbed CO_2_ (
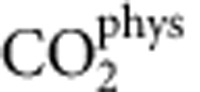
). Concomitantly, a strong band at 1,695 cm^−1^ transiently appears along with less intense bands at 1,547, 1,402 and 1,330 cm^−1^.

As can be seen in Fig. 2b the bands due to CO adsorbed on metallic Pt have little relation to the evolution of gas phase CO_2_ (CO_2(g)_): their temporal character indicates little more than adsorption/desorption processes related to the presence in this sample of metallic Pt nanoparticles. However, it may be noted that the lower wavenumber bands, and that due to 
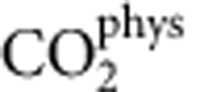
, only appear in the oxygen cycle when the removal of linear and bridged, adsorbed CO species starts to become appreciable. This shows that the reserve of ‘inactive' CO adsorbed on metallic Pt nanoparticles is the source through which the low-temperature active phase of Pt in this system is replenished and can convert CO to CO_2(g)_ in the absence of gas phase CO. This can occur as a result of CO desorption being mediated by physisorbed precursor states^19^,^20^. At ambient temperature, these have an appreciable lifetime and can therefore search out the active Pt centres required for reaction. The low-temperature catalytic production of CO_2_ observed therefore requires a communication between reduced nanoparticles and other highly dispersed, oxidic Pt centres.

The bands at lower wavenumber all show similar, although not identical, profiles that correlate well with CO_2_ production. The strongest correlation in both halves of the cycle (both in MS and DRIFTS via the 
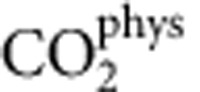
 band at 2,345 cm^−1^) is found for the band at *ca*. 1,695 cm^−1^. This principal band is very similar to that calculated by Moses-Debusk *et al.*^17^ for bidentate carbonates formed at oxidized and atomic Pt centres, lying midway between that calculated for a Pt(CO_3_) species (1,730 cm^−1^) and that observed by experiment (1,659 cm^−1^–0.18 wt% Pt and 1,637 cm^−1^–1 wt% Pt)^17^.

Although the summation of the evidence derived from MS and DRIFTS strongly suggests that a single Pt carbonate species is responsible for the majority of the room-temperature production of CO_2_, it would be remiss of us not to note that the number of bands, however weak, observed to correlate with the CO_2_ production would indicate the presence of more than a single carbonate species. However, and in addition to the negligible activity of an unloaded alumina support (Fig. 1c, red line), we may exclude the possibility that carbonates formed at the Al_2_O_3_ surface are participating in the chemistry shown in Fig. 2. Although such species show infrared absorption in the region of interest^21^,^22^ (Fig. 2b), none of them, by way of band position, combinations of bands and their expected relative intensities^22^, explains our observations. For instance, Al bicarbonates (1,655 cm^−1^)^21^,^22^ and bidentate carbonates (1,660–1,730 cm^−1^)^21^ always show bands in the 1,200–1,300 cm^−1^ region that should be detectable if they were contributing significantly to the chemistry^21^,^22^. Similarly, Al monodentate carbonates show spectral features only between 1,400 and 1,650 cm^−1^ (refs 21, 22). In this region, however, only the weak band at 1,402 cm^−1^ shows evidence for a diminution after its formation (although, by and large, it persists) and one that is very much slower than the majority CO_2_ production. As such, it cannot be deemed responsible for the majority CO turnover we observe that is far more highly correlated to the much stronger band at 1,695 cm^−1^.

Therefore, the above demonstrates that Pt carbonates are intrinsically capable of forming rapidly under CO and converting that CO to CO_2_ at 298 K. It also shows that re-oxidation of the Pt sites responsible for this chemistry is much slower and acts to limit the overall efficiency of this process.

### XAFS during periodic CO oxidation on 5 wt% Pt/Al_2_O_3_ at 298 K

**Figure 4:**
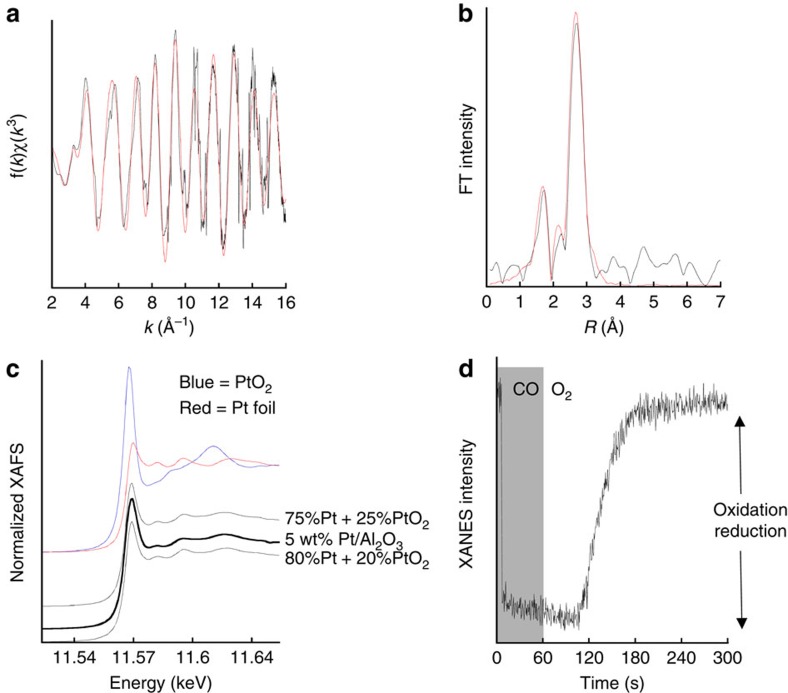
XAFS during periodic redox operation at 298 K. (**a**) *k*^3^-weighted EXAFS derived *in situ* from the 5 wt% Pt/Al_2_O_3_ catalyst along with theoretical fit (red); (**b**) the corresponding Fourier transform (again with the theoretical fit in red). (**c**) Comparison of Pt L_3_ edge X-ray absorption near edge structure from this sample with that from Pt foil (red) and PtO_2_ (blue). The sample spectrum is also compared with linear combinations (80% Pt^0^ and 75% Pt^0^ as indicated) of these two reference spectra. (**d**) The temporal variation observed in the Pt L_3_ white line intensity during exposure of the catalyst to 5 vol% CO/Ar and then 21 vol% O_2_/Ar.

DRIFTS and MS analyses tell us little about the Pt itself during these events. Importantly, these measurements cannot discriminate between a direct re-oxidation of isolated Pt centres that is subject to a relatively high activation energy—as considered by Moses-DeBusk *et al.*^17^—or whether, and as might be implied by the DRIFTS, desorption of molecular CO from metallic Pt nanoparticles has to occur first. To resolve this issue we therefore conducted static and time-resolved X-ray absorption fine structure (XAFS) spectroscopy at the Pt L_3_ edge under identical experimental conditions. Some results of this are shown in Fig. 4.

Figure 4a shows the k-weighted extended XAFS (EXAFS) obtained from the 5 wt% Pt/Al_2_O_3_ catalyst, whereas Fig. 4b shows the corresponding Fourier transform. The results of best fitting the EXAFS spectrum are given in Table 1. These measurements show that, as might be deduced from the dominance of linear and bridging CO bands in DRIFTS, the Pt in this sample principally comprises metallic nanoparticles. This is verified for a fresh sample by transmission electron microscopy (TEM, Supplementary Fig. 5). However, alongside this, and to a first approximation (Fig. 4c), a significant proportion (20–25%) of the Pt is present in a Pt (IV) oxidation state that would correspond to the relatively large, low Z (oxygen) coordination detected by the EXAFS (Fig. 4a,b and and Table 1). All or a portion of this oxidized Pt may correspond to the smaller Pt entities unambiguously detected by high-angle annular dark-field scanning TEM analysis (Supplementary Fig. 6). Moreover, the spatial proximity of these species to the reduced Pt nanoparticles makes the required molecular communication between these two types of Pt plausible.

Figure 4d shows the behaviour of the Pt during room-temperature exposure to CO and then 21 vol% O_2_/Ar, from the perspective of the height of the Pt L_3_ edge white line. This reveals that the reduction of the Pt—corresponding with the onset of CO_2_ production in the CO cycle—is extremely rapid and essentially complete in <10 s. On returning the sample to the oxidizing flow we clearly see that re-oxidation of the active Pt is much slower and subject to an induction time that matches well with the observations made using DRIFTS. From this we may conclude that re-oxidation of the carbonate precursor requires dissociation of O_2_ at the metallic sites, and that this can only occur as and when the linear and bridging CO species start to desorb.

## Discussion

The combined (DRIFTS, MS and time-resolved XAFS) evidence obtained allows us to derive a room-temperature cyclic mechanism, involving both Pt carbonates and Pt nanoparticles for the CO oxidation under the periodic conditions of operation employed.

Under CO:


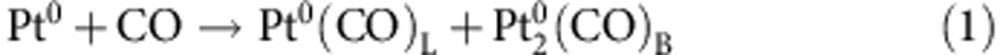



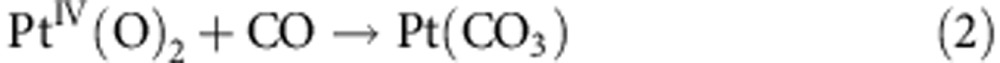



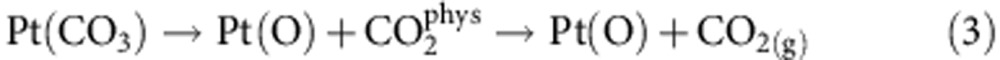


Under O_2_:


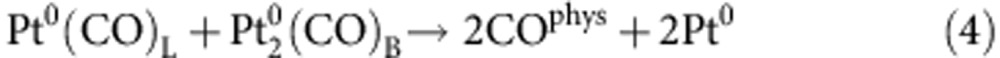



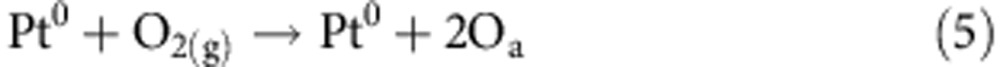



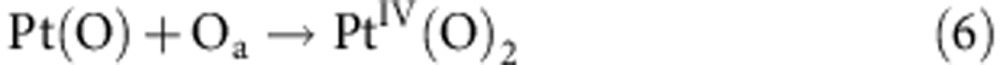



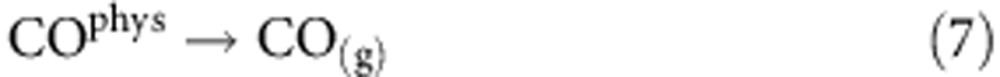



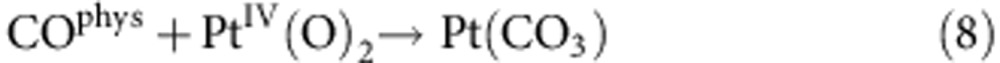



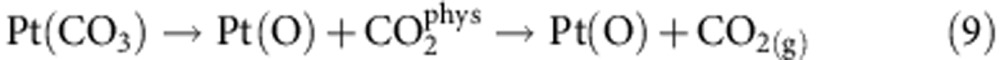



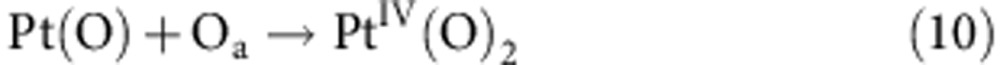


where Pt^0^ is the metallic platinum, Pt^0^(CO)_L_ is the CO adsorbed in linear geometry on metallic Pt, 
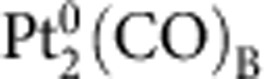
 is the CO adsorbed in twofold bridge geometry on metallic Pt, Pt(CO_3_) is the platinum carbonate intermediate, Pt^IV^(O)_2_ is the isolated oxidic platinum species, O_a_ is the adsorbed oxygen, Pt(O) is the product of carbonate decomposition, 
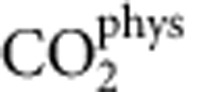
 and CO^phys^ are physisorbed CO_2_ and CO, respectively, and O_2(g)_, CO_2(g)_ and CO_(g)_ are gas phase species.

Within this mechanism, the oxidative regeneration of the precursor to carbonate formation, equation (6), limits the reformation of the carbonates in oxidizing conditions. However, the combined evidence suggests that this is mediated via the Pt^0^ nanoparticles and therefore can only happen once free sites are created on the Pt nanoparticles rather than through a direct dissociation of O_2_ at the precursor sites to the carbonates. As such, we suggest that it is CO desorption (equation (4)) that effectively limits the efficacy of this reactive pathway. In this respect, our mechanism differs from that of Moses-DeBusk *et al.*^17^ who considered only a direct dissociation of O_2_ by atomically dispersed Pt precursors to the carbonate species.

As a result, and in respect of steady-state operation, our room-temperature mechanism still suffers from the poisoning effect of CO that hobbles the classic LH mechanism. Indeed, steady-state operation (Supplementary Fig. 2) under a 4O_2_:CO flow leads to a very small, but non-zero, production of CO_2_ at room temperature; at present, it is only through adopting a periodic operation that this reactive impasse may be, to some degree, circumvented.

A priori we cannot definitively say where these carbonates actually reside. It is clear from our experimental methodology that the oxidized Pt entities that lead to their formation can survive reduction in H_2_/Ar to at least 573 K. This characteristic mitigates against their existence within a commensurate and reactive surface oxide in the manner proposed by Ackermann *et al.*^12^ Instead, in this respect we would tend to concur with the propositions of Moses-DeBusk *et al.*^17^ who only considered atomically dispersed oxidic Pt centres adsorbed on alumina surfaces. Further, high-angle annular dark-field scanning TEM measurements (Supplementary Fig. 6) also support this view and are consistent with recent works that have shown that similar atomic or quasi atomically adsorbed Pt and Pd species can be highly reactive for oxidation reactions^23^,^24^. Although we cannot rule out a role for interfacial Pt-O-Al sites, these would not be sufficient (based on the particle size distribution from TEM, see Supplementary Fig. 5) to yield the *ca*. 10–20% of active Pt that our results indicate are mediating carbonate formation and CO turnover in the current case.

To summarize, we have demonstrated that room-temperature CO oxidation over Pt/Al_2_O_3_ is feasible and mediated by oxidized, isolated and reduction-resistant Pt centres that may form reactive carbonates from CO and be re-oxidized under O_2_. Using periodic operation we have shown that this process is quantitatively reversible and can form a catalytic cycle. At present, the reactive Pt centres necessary for this low-temperature conversion exist as a minority species within the conventional catalyst used for these studies: most of the Pt (estimated to be *ca*. ≥80%) is not directly active but does have a role to play in completing the catalytic cycle. We have shown that at room temperature metallic nanoparticulate Pt may act as a reservoir for CO, which may be transferred to the minority quasi atomic oxidic Pt phase, and converted to CO_2_. The rate-limiting re-oxidation of the carbonate precursor sites (Pt(O)) also requires the presence of Pt nanoparticles that can facilitate O_2_ dissociation and oxygen transfer to these sites.

As such, this new pathway for achieving low-temperature CO oxidation immediately poses a number of further challenges to material synthesis and fundamental understanding. Foremost among these is the fundamental question of whether a direct oxidation of the precursor to the Pt carbonates can be attained and how we may promote CO desorption from the Pt nanoparticles to permit more efficient O_2_ dissociation. Second would be to understand, from a material synthesis perspective, how to optimize the levels of such carbonate forming Pt species on Al_2_O_3_ from *ca.* 10–20% minority to a working majority.

## Methods

### Infrared spectroscopy

Combined DRIFTS/MS experiments were carried at the Swiss Light Source using a Bruker Vertex 80V infrared spectrometer fitted with a narrow-band high-sensitivity mercury cadmium telluride (MCT) detector and with a Pfeiffer mass spectrometer. DRIFTS spectra were collected with a time resolution of 0.433 s and 2 cm^−1^ resolution.

The *in-situ* cell used for these experiments was that recently described by Chiarello *et al.*^25^ for transient experimentation and was windowed with CaF_2_. This cell was connected to a gas handling system equipped with fast solenoid switching valves (Parker) controlled using the infrared spectrometer. The reactor exit was connected to a Pfeiffer mass spectrometer that recorded both the sample temperature and the gas switching events alongside a range of masses compatible with the experiments being made.

Gas flows (50 ml min^−1^) were controlled by Bronkhorst mass flow controllers with a 2-bar pressure of gases behind the controllers themselves. This set-up allowed the purging of the system with Ar, then reduction using 5 vol% H_2_/Ar and, finally, gas switching experiments between 5 vol%CO/Ar and 21 vol%O_2_/Ar.

In the experiments described, 25–30 mg of the 5 wt% Pt/Al_2_O_3_ catalyst (Type-94, Johnson Matthey) was loaded into the DRIFTS cell. After purging in Ar, this sample was then reduced under 5 vol% H_2_/Ar to 573 K under a linear heating ramp (10 K min^−1^) and then held at 573 K for 30 min. The sample was then cooled under 5 vol% H_2_/Ar to 323 K whereon the reducing flow was substituted for flowing Ar once more. At 298 K, the sample was then exposed to 5 vol% CO/Ar for 52 s before the flow is switched to 21 vol% O_2_/He for 208 s, whereas DRIFTS and MS data were collected. This experiment was then repeated for a total of 16 CO/O_2_ cycles.

Subsequently, the sample was then exposed to a 4O_2_:1CO reaction mixture (again 50 ml min^−1^ total flow, 4.2 × 10^17^ molecules CO per second) and the temperature incremented until light-off of conventional CO oxidation catalysis was initiated (*T*∼423 K). At each temperature, the DRIFTS and MS data were collected, with the latter being used to calibrate the CO_2_ response in terms of the overall CO conversions.

Having conducted this calibration experiment, the sample was re-cooled under the reaction mixture, purged again before a second CO/O_2_ switching experiment was conducted and again DRIFTS and MS data simultaneously acquired. It is the results of this second switching experiment that are reported in Figs 1, 2, 3. We note that both first and second switching experiments, as well as the 16 cycle experiment, return the same global results save for some minor differences in overall CO_2_ production. In addition, although not shown here, essentially the same results can be achieved without any pre-reduction of the catalyst and the species responsible for the chemistry we have reported are present in the as-received catalyst.

### X-ray absorption spectroscopy

Pt L_3_ edge XAFS was collected in transmission mode at the SuperXAS beamline at the Swiss Light source (Villigen, Switzerland) using a newly installed fast, Si (111) channel cut monochromator system coupled to gridded N_2_-filled ionization chambers for detection. This bidirectional scanning system was operated at 2 Hz, yielding four spectra per second. The static and reference spectra are obtained as averages over 3 min of acquisition.

The *in-situ* time-resolved X-ray absorption near edge structure data were extracted from individual (250 ms time resolution) spectra during CO switching from 5 vol% CO/Ar and 21 vol% O_2_/Ar, and using the same sample environment as for the DRIFT/MS. Online MS was recorded as for the DRIFTS-based measurements. Data reduction was made using PAXAS^26^ and analysis of the EXAFS using EXCURV^27^.

## Additional information

**How to cite this article:** Newton, M. A. *et al.* Room-temperature carbon monoxide oxidation by oxygen over Pt/Al_2_O_3_ mediated by reactive platinum carbonates. *Nat. Commun.* 6:8675 doi: 10.1038/ncomms9675 (2015).

## Supplementary Material

Supplementary Information Supplementary Figures 1-6, Supplementary Discussion and Supplementary References

## Figures and Tables

**Table 1:** Best-fit parameters obtained for the 5 wt% Pt/Al_2_O_3_ sample measured *in situ* at room temperature and after reduction at 573 K.

**Element**	CN*	***R*** **(Å)**†	**DW (2*****σ***^2^**/(Å**^−2^**)**‡
Pt	8	2.75	0.014
O	1.9	2.00	0.007

EXAFS, extended X-ray absorption fine structure.

*E*_F_=−10.31=the edge position relative to the vacuum zero (Fermi energy).

*R*%=33.37=(∫[*χ*^T^−*χ*^E^]*k*^3^dk/[*χ*^E^]*k*^3^dk) × 100%, where *χ*^T^ and *χ*^E^ are the theoretical and experimental EXAFS and *k* is the photoelectron wave vector.

The Debye–Waller factor=2*σ*^2^, where *σ* is the root mean square internuclear separation.

Other parameters: Attentuation factor (AFAC), related to the proportion of electrons performing an EXAFS-type scatter on absorption, is 0.875. Structural data were obtained by fitting the EXAFS in *k* space in the range: *k*=2.5–16.5 Å^−1^.

^*^Coordination number (±*ca*. 10% stated value).

^†^Distance of scatterer atom from central atom (±*ca*. 1.5% stated value).

^‡^Debye–Waller factor.
