# Supplementary material for: Room-temperature carbon monoxide oxidation by oxygen over Pt/Al2O3 mediated by reactive platinum carbonates
Source: Nat Commun. 2015 Oct 22;6:8675. doi: 10.1038/ncomms9675 (PMC4846310; doi:10.1038/ncomms9675)
Supplement: Supplementary Information — Supplementary Figures 1-6, Supplementary Discussion and Supplementary References [file ncomms9675-s1.pdf]

Supplementary information

Supplementary Figures

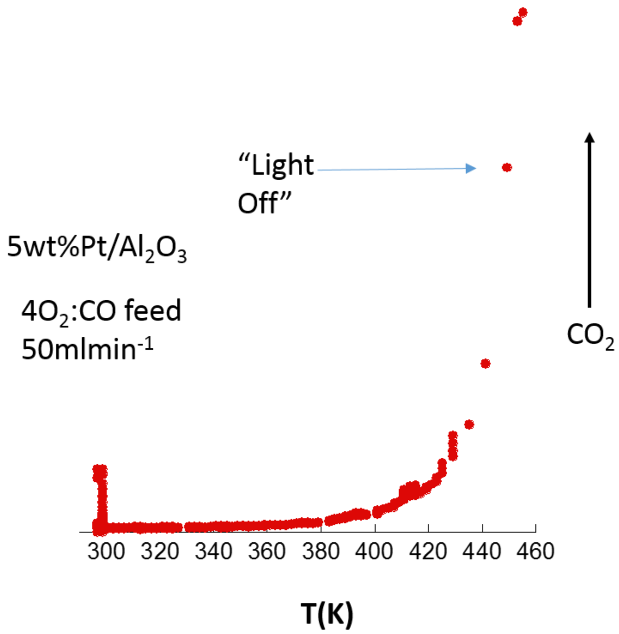

Supplementary Figure 1. CO_2 "light off" curve obtained from the 5 wt% Pt/Al_2O_3 catalyst obtained through heating the catalyst under a 50 ml.min^{-1} flow of a $4O_2$:CO feed. The transient burst of CO_2 at 298 K occurs when the switch is made between the reactive feed and 50 ml.min^{-1} Ar.

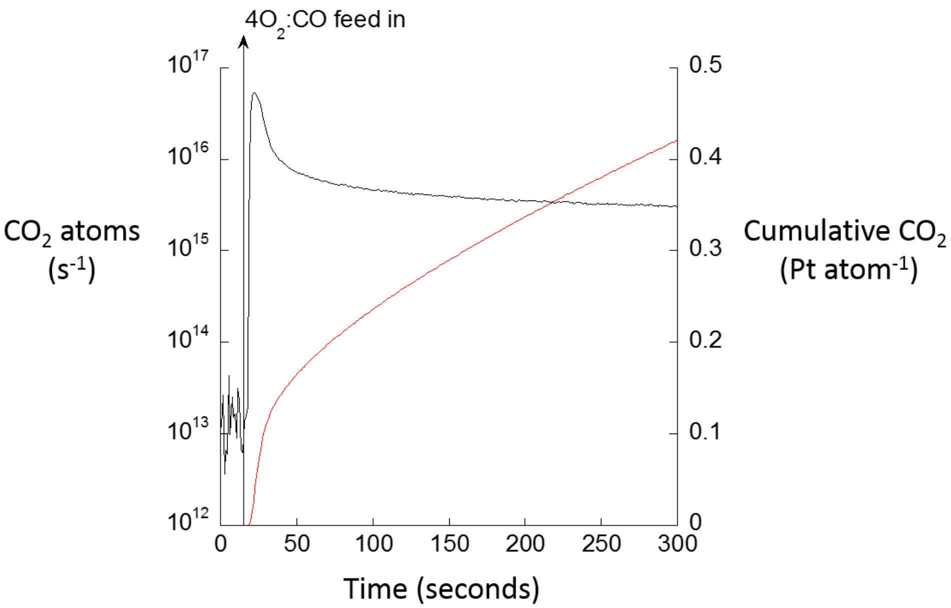

Supplementary Figure 2. CO_2 evolution resulting from a switch of feed from flowing Ar to a $4O_2$: CO at 298 K (via à vis Figure 1, main paper). Left axis: CO_2 molecules s^{-1}. 10^{13} represents the background level of CO inherent to the mass spectrometer vacuum system. Right axis: the cumulative CO_2 production observed in terms of the total number of Pt atoms in the sample bed.

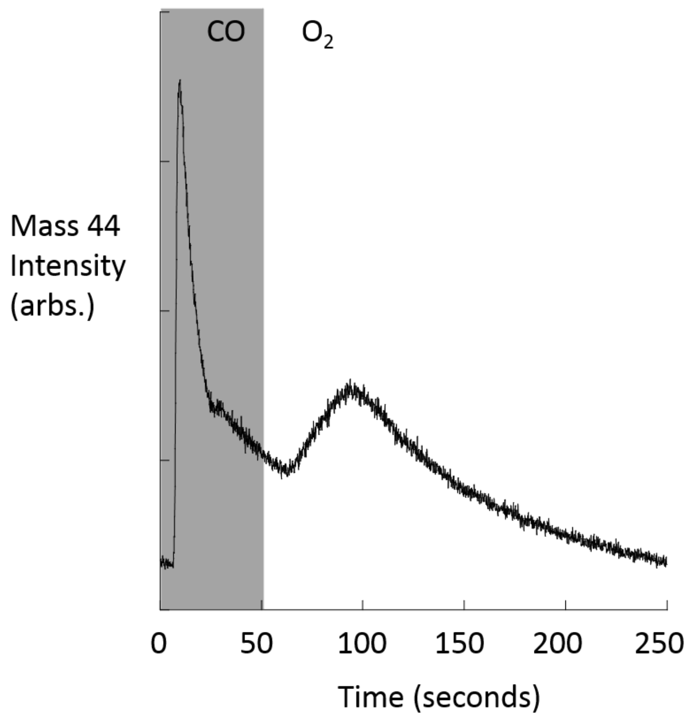

Supplementary Figure 3. CO_2 evolution observed over a 2 wt% Pt/Al_2O_3 catalyst (83 mg; Umicore) during cycling between CO and O_2 at 308 K and with timings similar to that shown for the T-94 catalyst (26 mg; Johnson-Matthey) in the main paper.

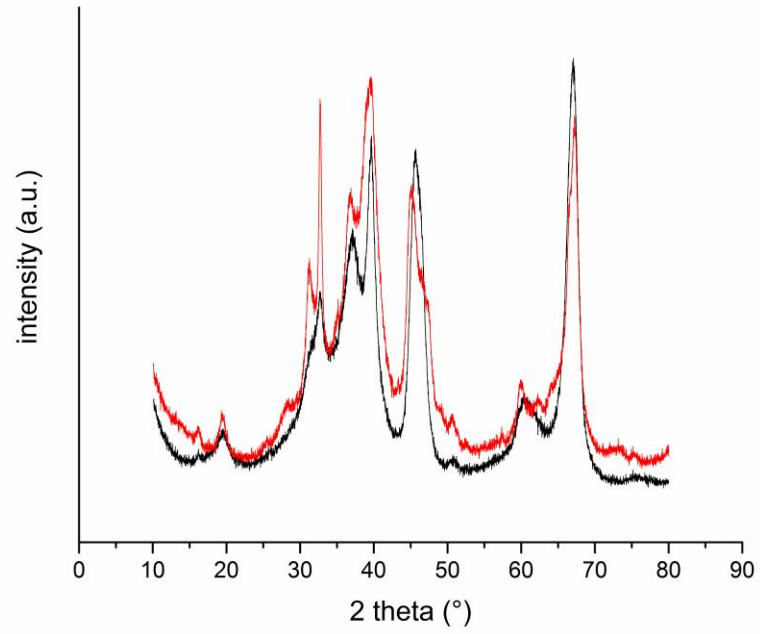

Supplementary Figure 4. XRD patterns of 5 wt% Pt/Al_2O_3 (Type-94, red) and 2 wt% Pt/Al_2O_3 (black).

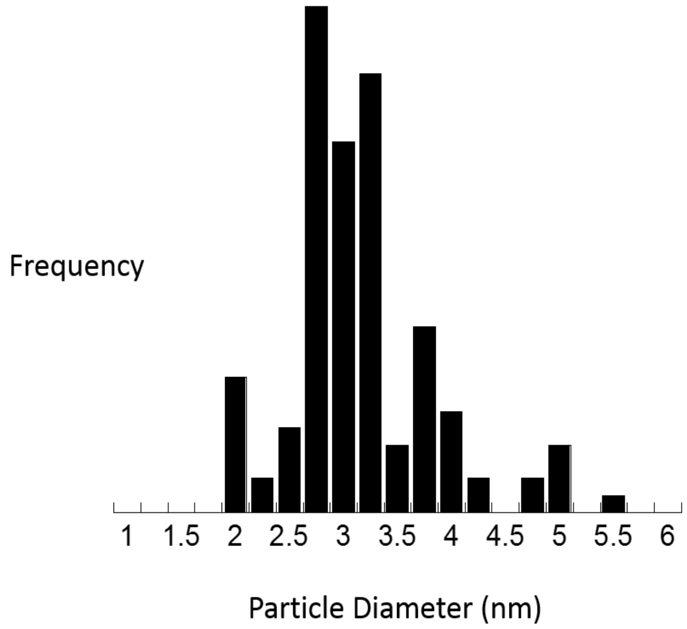

Supplementary Figure 5. TEM particle size distribution obtained from the Type-94 5 wt% Pt/Al_2O_3 catalyst.

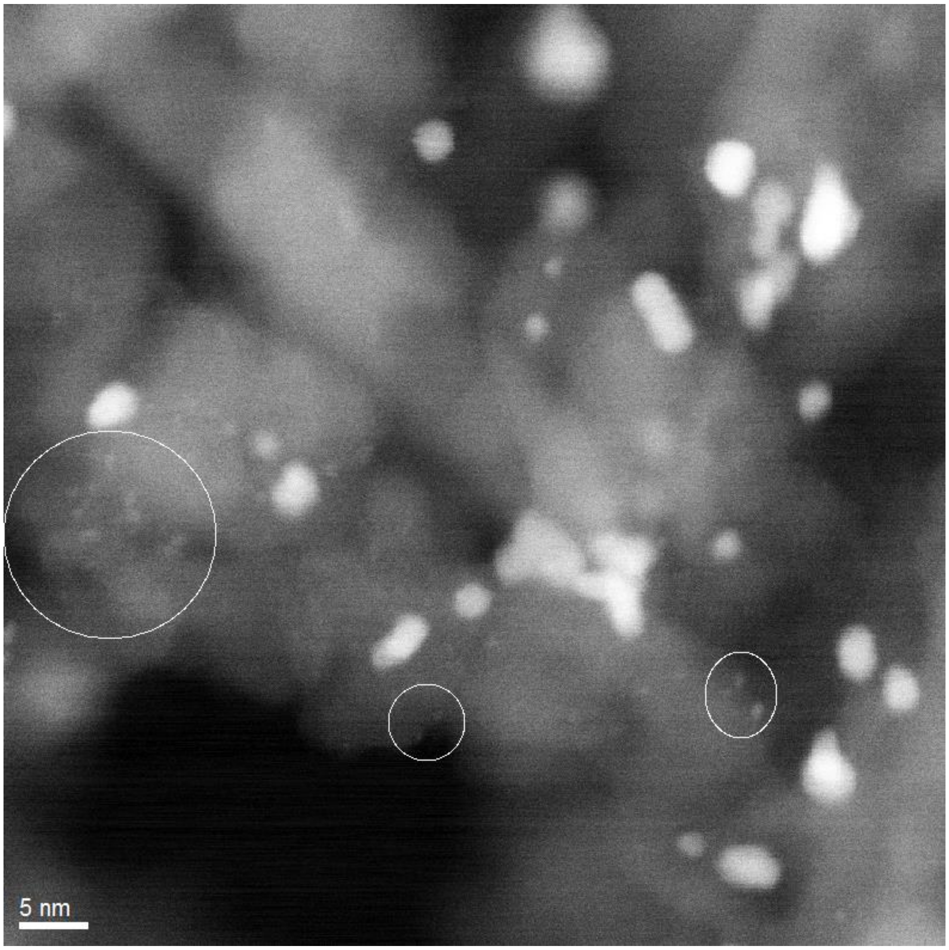

Supplementary Figure 6. HAADF-STEM image of the Type-94 5 wt% Pt/Al_2O_3 catalyst. The bright objects are the metallic Pt component of this catalyst whilst the circled elements are the isolated Pt(IV) centres indicated to be present from XAFS and indicate to be the source of the room temperature CO oxidation activity observed.

Supplementary Discussion

Conventional "light off" and a test of steady state operation at room temperature

Supplementary Figure 1 shows the light off curve obtained from the Type-94 catalyst after in situ reduction at 573 K. As expected the 5 wt% Pt/Al_2O_3 catalyst used in the current study shows a very conventional light off curve - with conventional catalytic CO oxidation taking off at ca. 450 K- as it is heated under a $4O_2$:CO feed. Supplementary Figure 2 shows CO_2 production observed for a period of ca. 300 s following a switch from 50 ml.min^{-1} Ar to a 50 ml.min^{-1} ($4O_2$:1CO) feed at 298 K. These results show that long after the initial transient pulse of CO_2 due to the formation and reaction of Pt carbonate species the level of CO_2 production does not return to background levels. Instead it persists at a lower level.

Within the mechanism postulated in the main paper we can state therefore that this catalysis is active under steady state conditions. Equally, however, the rate at which oxygen may be supplied to the precursor sites to the carbonate (either directly at these sites or as the result of dissociation by the metallic Pt nanoparticles and then oxygen transfer) is only sufficient to maintain this catalysis at a much lower level than is desirable. Therefore, along with optimizing the levels of the highly dispersed and oxidic Pt species required for carbonate formation, finding a more efficient route to their regeneration would seem to be, on a more fundamental level, the key to increasing the efficiency of this chemistry.

Reactivity of another Pt/Al_2O_3 catalyst: 2 wt% Pt/Al_2O_3

A second catalyst (kindly supplied by Umicore) was also investigated for room temperature CO oxidation. This catalyst has been extensively characterized previously[1] and shown to be additive free and comprised of Pt supported upon γ-Al_2O_3. Supplementary Figure 3 shows the temporal variation in CO_2 production observed in the MS from this sample maintained at 308 K and subject to similar sequential pulsing of CO and O_2.

It is clear from this figure that a similar general chemistry can be seen to be at work in this catalyst as the Type-94. However, it can be shown that the general levels of CO_2 production per redox strike is somewhat lower than for the Type-94 with peak levels of CO_2 production in the CO strike, for example, amounting to ca. 50% of that observed for the Type-94. Whilst at first sight this might be thought to scale with the relative Pt loadings of the two catalysts (i.e. 5 versus 2 wt%) we may

equally note that this is achieved using 83 mg of catalyst rather than the 26 mg of the Type-94. That is to say the 2 wt% Pt/γ-Al_2O_3 system appears to be at least six times less active (per gram catalyst) for this low temperature chemistry than the Type-94.

As such, we might conclude that this behavior is most likely general to Pt/Al_2O_3 systems but that the extent to which any given catalyst might display it will be dependent on numerous factors, which at this point remain to be investigated within the general notion of optimization.

Characterisation

A number of measurements were undertaken to characterize the commercial catalyst used in this study. X-ray fluorescence measurements (Microeagle apparatus) showed, within the limits of detection of this technique, that the Type-94 catalyst is additive free and only contained Al, O, and Pt.

X-ray diffraction (XRD). Supplementary Figure 4 compares the XRD patterns obtained from the 5 wt% Pt/Al_2O_3 and the 2 wt% Pt/Al_2O_3 catalysts. The latter has been previously assessed and the dominant phase found to be γ-Al_2O_3.[1] The results indicate that the Type-94 is a mixture of γ-Al_2O_3 and θ-Al_2O_3.

X-ray absorption spectroscopy. The results of the EXAFS analysis (Table 1) are consistent with TEM (Supplementary Figure 5) though the average size of the Pt nanoparticles using this non-cumulant corrected analysis[2,3] will likely underestimate their dimensions relative to the microscopy (mean particle diameter = 3nm). Equally, however, both the XANES and the EXAFS show that there is in this catalyst a significant proportion of O coordination (CN = 1.9, EXAFS) and Pt that exists in a higher oxidation state (XANES). A first approximation based on comparing a linear combination Pt foil and PtO_2 spectra (Figure 4C in main paper) would suggest that, at most, this could represent 20-25% of the Pt^{IV} is present in the system. This analysis, however, takes no account of the effect of the nanoscale size of these particles on their white line intensity relative to a foil, which may be significant in this size regime.[4,5] As such this analysis must be regarded as yielding an over estimate of the true level of reactive and oxidised Pt present in the system.

Given these facts, our observation would be consistent with the quantification of CO_2 production shown in Figure 1 and that the fraction of oxidised Pt that can resist reduction by H_2 using the conditions applied during these experiments is less than 20% of the total Pt present.
We note that the relatively large negative value of E_f (Table 1) indicates that cumulant effects, that have not been taken into account in this analysis, are most likely present in this system.[2] However, this does not affect the conclusions drawn from the EXAFS, vis à vis the predominantly metallic nature of the Pt, and a level of O coordination that would indicate that there is a significant level of oxidized Pt present in this catalyst.

TEM derived particle size distribution for 5 wt% Pt/Al_2O_3. TEM measurements were carried out at the Centre for Advanced Microscopy, University of Reading, United Kingdom. The 5 wt% Pt/Al_2O_3 was dispersed in isopropanol and ground prior to being applied to a TEM grid. Images were collected using a Jeol 2010 microscope equipped (LaB_6 electron gun, 200 keV).
The Type-94 catalyst is characterized as having a mean Pt particle size of ca. 3 nm in diameter (Supplementary Figure 5). If we assume that these particles are of a hemispherical nature this would indicate that the average particle contains around 650 Pt atoms with a dispersion of ca 0.365. This translates to an average particle perimeter that, at best, might comprise ca. 5% of the total Pt present. However, as this distribution is slightly asymmetric, and weighted toward the larger end of the particle sizes present, these averages overestimate both the total dispersion and the fraction of Pt atoms that could exist at the edges of the particles and in contact with the support materials. If we account for this then the fraction of possible Pt atoms at the perimeter of the particles falls to ca. 2.0%. As such we judge that, though we cannot rule out the participation of peripheral (Pt-O-Al) sites in the chemistry we have investigated, they cannot themselves explain the activity observed.
The sum of the evidence therefore points to the active species as being predominantly atomically dispersed and in intimate contact with the Al_2O_3.

HAADF-STEM analysis of 5 wt% Pt/Al_2O_3. To complement the standard TEM, HAADF-STEM measurements were carried out at the Scientific Center for Optical and Electron Microscopy (ETH Zurich, Switzerland). For HAADF-STEM measurements, the sample was dispersed in ethanol and some droplets of the suspension were deposited on a lacey carbon foil

supported on a Cu grid (Okenshoji, Japan). Scanning transmission electron microscopy (STEM) investigations were performed on the aberration-corrected HD-2700CS (Hitachi; cold-field emitter), operated at an acceleration potential of 200 kV. A probe corrector (CEOS) is incorporated in the microscope column between the condenser lens and the probe-forming objective lens providing high-resolution capability. The beam diameter selected here was ca. 0.2 nm and the images were recorded with a high-angle annular dark field ((HAADF) detector providing atomic number (Z) contrast. Furthermore, a secondary electron (SE) detector is installed inside the column of the HD-2700CS microscope above the sample allowing studying the sample morphology as well. The images (1024 x 1024 pixels) were recorded with frame times of 10-20 s. An EDX spectrometer (EDAX) is attached to this microscope. An example is shown in Supplementary Figure 6.

HAADF-STEM images show that within this catalyst three distinct structural features can be discerned. Firstly is the highly dispersed Al_2O_3 phase itself. Upon this phase some bright objects with apparent dimensions in the range of ca. 2-6 nm are found; EDX analysis confirms these as the nanoparticulate and metallic Pt observed in both the EXAFS and conventional TEM. Finally some small objects that have a decreased contrast relative to the Al_2O_3 – in the figure these have been circled to guide the eye. These elements appear to have a dimensionality of < 1nm and represent the isolated, or quasi-isolated atomic Pt(IV) centres that XANES has indicated are present in this system and that we identify as the reactive elements that we have observed working at room temperature in this study.

Supplementary References

(1) Matam S. K., Kondratenko E. V., Aguirre M. H., Hug, P., Rentsch D., Winkler A., Weidenkaff A., Ferri D., The impact of aging environment on the evolution of Al_2O_3 supported Pt particles and their NO oxidation activity, *Appl. Catal. B*, **129**, 214-224, (2013)

(2) Clausen B. S., Norskov J. K., Asymmetric pair distribution functions in catalysts, *Top. Catal.*, **10**, 221-230, (2000)

(3) Bus E., Miller J. T., Kropf A. J., Prins R., van Bokhoven J. A., Analysis of in situ EXAFS data of supported metal catalysts using the third and fourth cumulant, *Phys. Chem. Chem. Phys.*, **8**, 3248-3258, (2006)

(4) Bazin D., Sayers D., Rehr J. J., Mottet C., Numerical simulation of the platinum L-III edge white line relative to nanometer scale clusters, *J. Phys. Chem B.*, **101**, 5332-5336, (1997)

(5) Nagai Y., Dohmae K., Ikeda Y., Takagi N., Tanabe T., Hara N., Guilera G., Pascarelli S., Newton M. A., Kuno O., Jiang H., Matsumoto S., In situ redispersion of Platinum autoexhaust catalysts: an online approach to increasing catalyst lifetimes?, *Angew. Chem. Int. Ed.*, **48**, 9303-9306 (2008)
